# Obesity and Risk of Diabetes Mellitus by Menopausal Status: A Nationwide Cohort Study

**DOI:** 10.3390/jcm10215189

**Published:** 2021-11-06

**Authors:** Han Rim Lee, Jungeun Shin, Kyungdo Han, Jiwon Chang, Su-Min Jeong, Seung Joo Chon, Soo Jung Choi, Dong Wook Shin

**Affiliations:** 1 Department of Family Medicine & Supportive Care Center, Samsung Medical Center, Sungkyunkwan University School of Medicine, Seoul 06351, Korea; lhl1221@gmail.com (H.R.L.); lovevsv@gmail.com (J.S.); wldnjs5353@gmail.com (J.C.); smjeong.fm@gmail.com (S.-M.J.); 2 International Healthcare Center, Samsung Medical Center, Seoul 06351, Korea; 3 Department of Statistics and Actuarial Science, Soongsil University, Seoul 06978, Korea; hkd917@naver.com; 4 Department of Obstetrics and Gynecology, Gachon University Gil Medical Center, Incheon 21556, Korea; sjchon@gilhospital.com; 5 Department of Family Medicine, Gachon University Gil Medical Center, Incheon 21556, Korea; 6 Department of Digital Health, Samsung Advanced Institute for Health Science & Technology, Sungkyunkwan University, Seoul 06351, Korea

**Keywords:** diabetes mellitus, obesity, menopause

## Abstract

Although both obesity and menopause are associated with increased risk of diabetes mellitus (DM), the association between obesity and DM according to menopausal status remains uncertain. Therefore, we conducted a study to examine the relationship between obesity and incidence of diabetes mellitus (DM) in premenopausal and postmenopausal women. Total of 926,196 premenopausal and 1,193,881 postmenopausal women who underwent health examinations from 2009 to 2014 were identified using the database of the Korean National Health Insurance Service. We compared the incidence and risk of DM according to body mass index (BMI) and waist circumference (WC) in the two groups of women. Cox proportional hazards analyses were performed to evaluate the association between the presence of obesity and risk of DM according to menopausal state. During the 7.8-year follow-up period, 37,736 (4.1%) premenopausal women and 121,102 (10.1%) postmenopausal women were diagnosed with DM. Compared to the reference group (BMI 18.5–23), a stronger association between obesity and risk of DM was observed in both pre- and postmenopausal women: multivariable-adjusted hazard ratios and 95% confidence intervals for BMI subgroups <18.5, 23–25, 25–30, and >30 were 0.62 (0.54, 0.70), 1.91 (1.85, 1.97), 3.38 (3.28, 3.47), and 6.25 (6.02, 6.48), respectively (*p* trend < 0.001) in premenopausal women and 0.87 (0.82, 0.92), 1.44 (1.41, 1.46), 2.00 (1.97, 2.03), and 2.96 (2.89, 3.02) in postmenopausal women (*p* trend < 0.001, *p*-interaction < 0.001). A similar trend was observed for WC. Subgroup analyses of women aged 45 to 55 also showed a stronger association with DM in premenopausal than in postmenopausal women. In conclusion, the association between obesity and DM was stronger in premenopausal women than in postmenopausal women. As estrogens are synthesized in adipose tissue by aromatization of androgens after menopause, increased estrogen levels in obese postmenopausal might have a protective effect against DM.

## 1. Introduction

Diabetes mellitus (DM) is a major public-health problem, with a global prevalence of 9.3% (463 million people) in 2019 that is expected to reach 10.2% (578 million people) in 2030 [1]. Obesity, the most prominent risk factor of DM and that leads to the development of insulin resistance, is more common in women than men [2,3,4,5,6]. Women gain about 0.7 kg/year on average, independent of race [7,8] and more women are overweight or obese after age 45 years, whether more males tend to be overweight at younger age [9].

On the other hand, menopause is a potential risk factor for the development of DM, likely due to a reduction in circulating estrogens [10]. The Study of Women’s Health Across the Nation (SWAN) suggested that lower E2 concentrations resulted in a 47% higher risk of type 2 DM during the menopausal transition [11]. The European Prospective Investigation into Cancer (EPIC)-InterAct study suggested that menopause before the age of 40 years was associated with a 32% greater risk of type 2 DM [12]. A previous meta-analysis reported that levels of fasting glucose (weighted mean difference [WMD] 4.64, 95% confidence interval [CI] 3.94–5.33) and fasting insulin (WMD 20.88, 95% CI 2.12–39.65) were both increased after menopause [13].

Randolph et al. reported that obese women had lower pre-menopausal estradiol level, which is sampled on day 2–5 of a spontaneous menstrual cycle, but higher post-menopausal estradiol level compared to non-obese women [14]. However, the association between obesity and DM according to menopausal status remains unclear. One US study combining the Health Professionals Follow-up Study and the Nurses’ Health Study found the association between body mass index (BMI) and risk of DM to be significantly stronger among younger women (age < 60 years) compared to older women (age 60–69 years and age ≥ 70 years, *p* trend < 0.001) [15]. While that study included menopausal status as a covariate, the population was generally older (mean age ~ 64 years) and thus could not assess the relationship stratified by menopausal status.

Therefore, we conducted a retrospective cohort study to examine the association between obesity and risk of DM in pre- and postmenopausal women using a nationwide population-based data set in Korea.

## 2. Materials and Methods

### 2.1. Data Source and Study Setting

This study used data from the National Health Insurance Service (NHIS), a single government insurer that provides a mandatory universal insurance system that covers approximately 97% of the Korean population, while the remaining 3% with low income are covered by the Medical Aid program. Medical service providers submit claims including data on demographics, diagnoses, and medical treatment for reimbursement. In addition, the NHIS provides a biennial National Health Screening Program (NHSP) that includes screening for cardiovascular risk factors, as well as the National Cancer Screening Program (NCSP) that includes breast and cervical cancer screening for all women aged 40 and above [16]. Therefore, the NHIS database contains an eligibility database (age, sex, socioeconomic variables, etc.), a medical treatment database, and a health screening database (health examination results and questionnaires on lifestyle and behavior).

### 2.2. Study Population

**Figure 1:**
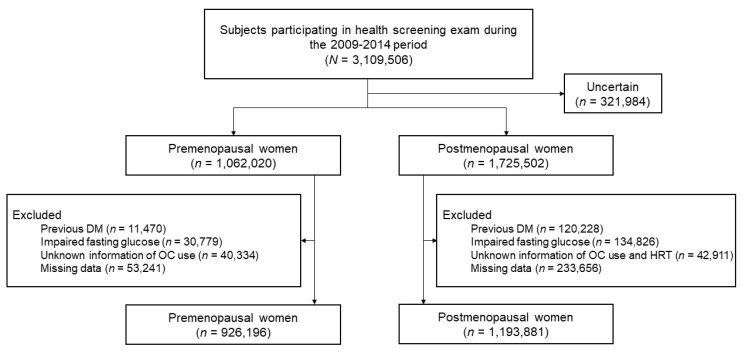
Flow chart of the study population.

Women aged 40 years or older in the NHIS database who had received NHSP and NCSP services from 1 January 2009 to 31 December 2014 were included. Menopausal status was ascertained by self-reported questionnaire. We excluded those with uncertain information on menopausal status (*n* = 321,984), those who were previously diagnosed with DM (*n* = 11,470 in premenopausal, *n* = 120,228 in postmenopausal women), and those with high fasting glucose (≥126 mg/dL) at the health examination (*n* = 30,779 in premenopausal, *n* = 134,826 in postmenopausal women). In addition, those with missing information on reproductive history (*n* = 40,334 in premenopausal, *n* = 42,911 in postmenopausal women) and those with other missing covariates (*n* = 53,241 in premenopausal, *n* = 233,656 in postmenopausal women) were excluded. Finally, a total of 2,120,077 individuals (926,196 premenopausal and 1,193,881 postmenopausal women) were included in the analysis (Figure 1).

### 2.3. BMI and WC

During the health examinations, height and weight were measured. BMI (kg/m^2^) was calculated using the individual’s body weight (kg) divided by the square of height (m^2^) and was categorized as low (<18.5), normal (reference, 18.5–22.9), overweight (23–24.9), obese (25–29.9), or severely obese (≥30) according to the Asia-Pacific BMI criteria established by the Western Pacific Region of the World Health Organization [17]. Waist circumference (WC) (cm) was measured at the midpoint between the lower margin of the last palpable rib and the top of the iliac crest [18] and was divided into 6 levels: <75, 75–79.9 (reference category), 80–84.9, 85–89.9, 90–94.9, and ≥95 cm.

### 2.4. Study Outcomes and Follow-Up

The primary endpoint of this study was newly diagnosed DM defined by ICD-10 codes for DM (E11-14) with a prescription history of hypoglycemic agents. The cohort was followed from the initial health check-up date to the date of incidence of DM, death, or until the end of the study (31 December 2017), whichever came first.

### 2.5. Covariates

Detailed information on health-related behavior and reproductive history were collected through self-reported questionnaires. Smoking status was categorized into three groups: never, former, and current smokers. Alcohol consumption was assessed as the amount of alcohol consumed per occasion and the frequency of alcohol intake per week and classified into non-, mild (<30 g/day), and heavy drinkers (≥30 g/day). Regular physical activity was defined as moderate severity physical activity for more than 30 min on more than 5 days per week over the past week. The age at menarche was categorized as ≤12, 13–14, 15–16, and >16 years, and the age at menopause was categorized as <40, 40–44, 45–49, 50–54, or ≥55 years. Participants’ reproductive history included parity (0, 1, or ≥2), total duration of breastfeeding (never, <6, 6–12, or ≥12 a total of months), duration of oral contraceptive use (never, <1, or ≥1 year), and duration of hormone replacement therapy (HRT) (never, <2, 2–5, or ≥5 years).

Comorbidities (e.g., hypertension, hyperlipidemia, or chronic kidney disease) were identified based on physician diagnosis or self-reported prescription medication history.

### 2.6. Statistical Analysis

Baseline characteristics were presented as mean ± standard deviation (SD) for continuous variables and number with proportion for categorical variables. Incidence rates of DM were estimated as the number of events per 1000 person-years. Cox proportional hazards analyses were performed to evaluate the associations between BMI, WC, and risk of DM by menopausal state. Model 1 was non adjusted, and Model 2 was adjusted for age, income, smoking, alcohol drinking, regular physical activity, and fasting glucose level. Model 3 was additionally adjusted for comorbidities. Finally, Model 4 was additionally adjusted for reproductive history (parity, duration of breastfeeding, duration of oral contraceptive use, age at menarche, age at menopause, duration of HRT).

As previous studies suggested that age-related factors can affect the association between obesity and DM risk, we stratified the analysis by age (40–49 years, 50–59 years, and ≥60 years) in all participants. As the association between obesity and DM risk could be driven by age rather than hormonal status, we conducted additional analyses in a narrow range of age (45–54 years) known as the perimenopausal transition.

Statistical analyses were performed using SAS version 9.4 (SAS Institute Inc., Cary, NC, USA), and a *p* value less than 0.05 was considered statistically significant.

## 3. Results

### 3.1. Baseline Characteristics of Study Subjects

Baseline characteristics of the study participants according to menopausal status are described in Table 1. During a mean follow-up of 7.8 years, 37,736 (4.1%) premenopausal women and 121,102 (10.1%) postmenopausal women were diagnosed with DM. Participants who were diagnosed with DM were more likely to be older (age 46.5 vs. 44.9 in premenopausal women, 63.1 vs. 61.0 in postmenopausal women) and obese (BMI 25.8 vs. 23.0 in premenopausal women, 25.4 vs. 23.9 in postmenopausal women) in each group according to menopausal status.

### 3.2. Associations of BMI and WC with Risk of DM

**Figure 2:**
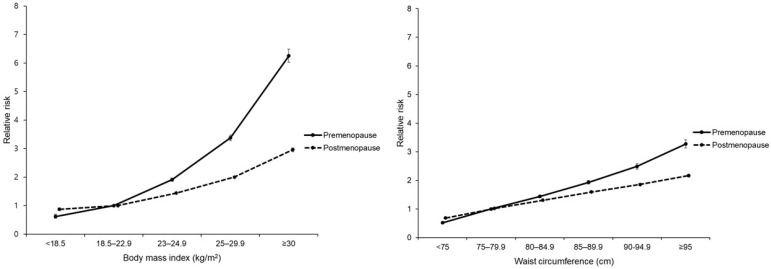
Diabetes incidence according to body mass index and waist circumference by menopausal status.

The incidence of DM was 2.07 and 7.28 per 1000 person-years in premenopausal women with normal BMI and postmenopausal women with normal BMI, respectively. Compared with the normal BMI group, adjusted hazard ratio [aHR; (95% CI)] for DM in the BMI <18.5, 23–25, 25–30, and >30 groups was 0.62 (0.54, 0.70), 1.91 (1.85, 1.97), 3.38 (3.28, 3.47), and 6.25 (6.02, 6.48) (*p* trend < 0.001), respectively, in premenopausal women and 0.87 (0.82, 0.92), 1.44 (1.41, 1.46), 2.00 (1.97, 2.00), and 2.96 (2.89, 3.02) in postmenopausal women (*p* trend < 0.001; *p*-for-interaction between BMI and menopause < 0.001). A similar trend was observed for WC in both premenopausal and postmenopausal women. However, there was some attenuation of the association between obesity indicators (BMI and WC) and DM risk in postmenopausal women compared with premenopausal women (Table 2 and Figure 2).

### 3.3. Analyses Stratified by Age Group

**Figure 3:**
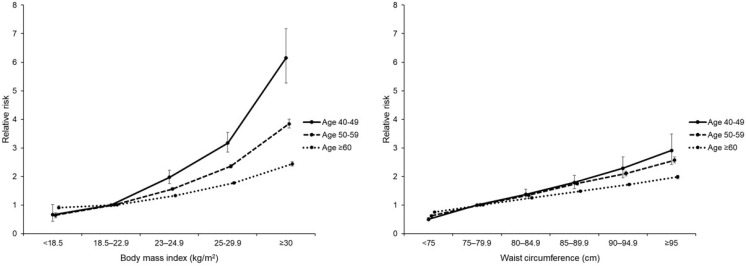
Diabetes incidence according to body mass index and waist circumference by age group.

Table 3 shows the associations of BMI and WC with DM risk stratified by age. Compared with a reference group (BMI 18.5–22.9 kg/m^2^, WC 75–79.9 cm), the aHRs (95% CI) for DM in the BMI <18.5, 23–25, 25–30, and >30 groups were 0.66 (0.43, 1.02), 1.97 (1.75, 2.22), 3.17 (2.85, 3.54), and 6.15 (5.27, 7.17), respectively, while those values in the WC <75, 80–84.9, 85–89.9, 90–94.9, and ≥95 groups were 0.50 (0.45, 0.57), 1.37 (1.22, 1.55), 1.79 (1.57, 2.04), 2.29 (1.95, 2.68), and 2.91 (2.42, 3.49) in women in their 40s (*p* trend < 0.001). In both groups of women aged 50–59 and ≥60s, aHR increased gradually as BMI and WC increased but not as precipitously as the values of those in their 40s (Figure 3).

### 3.4. Analyses with Transitional Age

**Figure 4:**
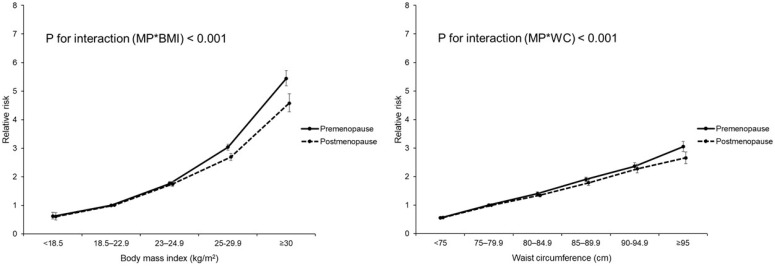
Diabetes incidence according to body mass index and waist circumference by menopausal status in 45–55-year-old women.

Among participants aged 45–54 years, aHR values for DM in the BMI <18.5, 23–25, 25–30, and >30 groups were 0.62 (0.52, 0.75), 1.76 (1.69, 1.83), 3.04 (2.93, 3.14), and 5.44 (5.18, 5.72) in premenopausal women and 0.60 (0.48, 0.74), 1.74 (1.66, 1.83), 2.69 (2.57, 2.81), and 4.57 (4.27, 4.90) in postmenopausal women, respectively (*p* trend < 0.001; *p*-for-interaction between BMI and menopause < 0.001). A similar trend was observed for WC in both premenopausal and postmenopausal women (*p* trend < 0.001; *p*-for-interaction between WC and menopause < 0.001) (Table 4 and Figure 4).

## 4. Discussion

In this study, we investigated the association between obesity as defined by indicators (BMI and WC) and risk of DM in premenopausal and postmenopausal women. We found that obesity was associated with increased risk of DM in both groups but with a stronger association in premenopausal women than in postmenopausal women. In sensitivity analysis with transitional aged women (45–54 years) considering an aging effect on DM incidence, a consistently stronger association was found in premenopausal women than postmenopausal women.

The increased risk of developing DM after menopause is due to a decrease in estrogen. Weight gain tends to accompany aging in a woman’s life cycle and has been suggested to be a result of decreasing estrogen level after menopause, as estrogen facilitates adipose tissue function and deposition [19]. Thus, menopause is followed by adipose tissue redistribution to visceral depots, which is associated with insulin resistance, while greater subcutaneous gluteal-femoral fat is associated with protection from metabolic syndrome [20].

On the other hand, the impact of body weight on DM risk differs in obese women depending on menopausal status. In premenopausal women, obesity has a direct inhibitory effect on estradiol production from the ovaries [21,22] while estrogens are synthesized in adipose tissue by aromatization of androgens after menopause [23]. Indeed, estradiol level is lower in obese women than non-obese women at premenopausal age but is higher in obese women of post-menopausal age [14]. The present study shows that the association between obesity and DM was more prominent in premenopausal women than in postmenopausal women. It seems that obesity before menopause increases the risk of DM by lowering the estrogen level in the body in addition to an increase in insulin resistance caused by obesity itself. On the contrary, obesity after menopause can increase the estrogen level in the body, providing a protective effect against DM.

The different tendencies of association between obesity and DM by menopausal status can be explained by the change of body composition during the menopause transition. During pre-menopause, fat mass tends to increase, while the proportion of lean mass decreases over time. However, in Chinese women, decreasing fat mass and increasing proportional lean mass were found after menopausal transition [24]. Therefore, high BMI might reflect excess fat mass, which contributes to the association between obesity and DM, in premenopausal women rather than postmenopausal women.

Our study results might be confounded as menopause is linked with aging itself. Therefore, we limited our analyses to women aged 45–54 years, when menopause generally occurs. Even after adjusting for age and other confounding factors, a stronger association was found in premenopausal women, supporting the main results of our study and the above explanations.

Clinical implications of our study are as follows. First, it is important to reduce obesity in premenopausal women. A previous study investigated the age differences between BMI and DM incidence and showed relatively higher risk in younger age [aHR (95% CI) 4.72 (1.79, 12.40) in 30–39 aged women, 1.54 (1.02, 2.33) in 50–59 aged women] [25]. The Asia Pacific Cohort Studies Collaboration also suggested stronger relationships between BMI and risk of DM in younger women (for each reduction in BMI of 2 kg/m^2^, 31% lower risk in group age ≤60, 19% lower risk in group age >70, and 27% lower risk in women compared to 23% lower risk in men) [26]. As diabetic women manifest heightened cardiovascular mortality compared to diabetic men, it is important to prevent DM in women [20,27]. Second, given that estrogen might have a protective effect, HRT might be helpful to prevent diabetes mellitus, especially in lean women. In the large Nurses’ Health Study, current users of hormonal therapy had reduced incidence of DM; relative risk 0.80 (0.67, 0.96) compared to non-users after adjusting for age and BMI [28]. The WHI and HERS trials also showed similar findings [29,30]. Our results provide additional evidence for the potential benefit of HRT in the prevention of DM and cardiovascular disease, although use of HRT should be based on the balance of benefits and risks.

This study has several limitations. First, we did not directly measure the fat mass, which was more strongly associated with the risk of DM than was BMI, although BMI typically is a good indicator of body fatness [31]. Second, as this study predominantly included people who underwent health examinations, participants tended to be healthier than the general population. Third, we obtained information about menopause based on a self-administered questionnaire, which might have caused misclassification. Fourth, as the study design was observational, it was not possible to distinguish the effects of age from those of menopause. Lastly, although we adjusted for known confounders, we cannot rule out unmeasured and residual confounding. Despite these limitations, this study included a large representative sample and used both BMI and WC indicators of obesity. To our knowledge, this is the first large cohort study to evaluate the association between obesity and risk of DM incidence separately in pre- and postmenopausal women.

In conclusion, this study found a stronger association between obesity and risk of DM among premenopausal women compared with postmenopausal women in the Korean population. Future studies are needed to better understand the precise mechanism of the different relationships between obesity and incidence of diabetes by menopausal status.

## Figures and Tables

**Table 1:** Baseline characteristics of the study population according to menopausal status.

	Premenopausal			Postmenopausal		
	DM			DM		
	No	Yes	*p*-Value	No	Yes	*p*-Value
	*n* = 888,460	*n* = 37,736		*n* = 1,072,779	*n* = 121,102	
	N (%)	N (%)		N (%)	N (%)	
**Age, mean (SD)**	44.91 (±3.9)	46.47 (±4.1)	<0.001	61 (±8.3)	63.06 (±7.9)	<0.001
**Body mass index, kg/m^2^,** **mean (SD)**	23.03 (±2.9)	25.81 (±3.6)	<0.001	23.88 (±3.0)	25.44 (±3.3)	<0.001
<18.5	26,271 (3.0)	237 (0.6)	<0.001	25,901 (2.4)	1346 (1.1)	<0.001
18.5–22.9	455,929 (51.3)	7929 (21.01)		400,471 (37.3)	24,883 (20.6)	
23–24.9	207,975 (23.4)	8598 (22.8)		288,280 (26.9)	29,625 (24.5)	
25–29.9	177,510 (20.0)	16,195 (42.9)		324,374 (30.2)	54,754 (45.2)	
≥30	20,775 (2.3)	4777 (12.7)		33,753 (3.2)	10,494 (8.7)	
**Waist circumference, cm**			<0.001			<0.001
<75	470,509 (53.0)	8121 (21.5)		314,484 (29.3)	15,666 (12.9)	
75–79.9	203,157 (22.9)	8472 (22.5)		256,275 (23.9)	22,281 (18.4)	
80–84.9	126,288 (14.2)	8838 (23.42)		247,831 (23.1)	31,458 (26.0)	
85–89.9	56,024 (6.3)	6116 (16.21)		146,726 (13.7)	25,296 (20.9)	
90–94.9	21,496 (2.4)	3445 (9.13)		71,134 (6.6)	15,821 (13.1)	
≥95	10,986 (1.2)	2744 (7.27)		36,329 (3.4)	10,580 (8.7)	
**Smoking status**			<0.001			<0.001
Never	844,864 (95.1)	35,395 (93.8)		1,034,949 (96.5)	115,434 (95.3)	
Ex-smoker	14,279 (1.6)	570 (1.5)		10,902 (1.0)	1428 (1.2)	
Current	29,317 (3.3)	1771 (4.7)		26,928 (2.5)	4240 (3.5)	
**Alcohol drinking**			<0.001			<0.001
Non	635,208 (71.5)	28,005 (74.2)		933,632 (87.0)	107,606 (88.9)	
Mild	243,387 (27.4)	9125 (24.2)		133,670 (12.5)	12,840 (10.6)	
Heavy	9865 (1.1)	606 (1.6)		5477 (0.5)	656 (0.5)	
**Regular physical activity**	153,878 (17.3)	6424 (17.0)	0.137	197,854 (18.4)	21,252 (17.6)	<0.001
**Income**			<0.001			0.135
1st quartile (lowest)	227,896 (25.7)	10,182 (27.0)		244,674 (22.8)	27,725 (22.9)	
2nd quartile	179,239 (20.2)	8385 (22.2)		199,691 (18.6)	22,568 (18.6)	
3rd quartile	195,797 (22.0)	8556 (22.7)		264,510 (24.7)	30,114 (24.9)	
4th quartile (highest)	285,528 (32.1)	10,613 (28.1)		363,904 (33.9)	40,695 (33.6)	
**Comorbid condition**						
Hypertension	111,075 (12.5)	12,204 (32.3)	<0.001	438,377 (40.9)	72,790 (60.1)	<0.001
Hyperlipidemia	86,935 (9.8)	8950 (23.7)	<0.001	322,240 (30.0)	52,569 (43.4)	<0.001
Chronic kidney disease	36,644 (4.1)	1706 (4.5)	0.002	113,384 (10.6)	16,155 (13.3)	<0.001
History of stroke	2037 (0.5)	122 (0.6)	0.03	12,511 (1.8)	2098 (2.4)	<0.001
History of heart disease	3903 (0.9)	348 (1.6)	<0.001	34,181 (4.9)	6793 (7.6)	<0.001
SBP, mmHg	116.7 (±14.1)	123.89 (±15.6)	<0.001	124.65 (±16.0)	129.01 (±16.1)	<0.001
DBP, mmHg	72.85 (±9.9)	77.37 (±10.5)	<0.001	76.59 (±10.1)	78.61 (±10.1)	<0.001
Fasting glucose, mg/dL	91.02 (±10.1)	101.4 (±12.5)	<0.001	92.94 (±10.6)	101.35 (±12.4)	<0.001
Total cholesterol, mg/dL	191.22 (±38.3)	204.78 (±41.0)	<0.001	208.5 (±43.2)	213.16 (±45.9)	<0.001
HDL, mg/dL	60.72 (±35.0)	55.8 (±32.1)	<0.001	58.45 (±35.7)	55.98 (±35.8)	<0.001
LDL, mg/dL	114.01 (±70.2)	123.07 (±60.3)	<0.001	128.07 (±68.0)	129.52 (±78.3)	<0.001
AST, mg/dL	20.33 (20.31–20.34)	22.81 (22.72–22.9)	<0.001	23.71 (23.7–23.72)	25.19 (25.14–25.24)	<0.001
ALT, mg/dL	16.35 (16.33–16.36)	22.08 (21.96–22.21)	<0.001	19.44 (19.42–19.46)	23.06 (22.99–23.12)	<0.001
rGTP, mg/dL	16.8 (16.78–16.81)	24.36 (24.21–24.51)	<0.001	19.62 (19.6–19.64)	25.08 (25–25.17)	<0.001
TG, mg/dL	86.23 (86.15–86.32)	125.55 (124.87–126.23)	<0.001	111.02 (110.91–111.12)	136.95 (136.55–137.34)	<0.001
**Age at menarche, years,** **mean (SD)**	15.09 (±1.7)	15.28 (±1.8)	<0.001	16.44 (±1.8)	16.58 (±1.8)	<0.001
**Age at menarche, years**			<0.001			<0.001
≤12	38,914 (4.4)	1678 (4.5)		10,589 (1.0)	1087 (0.9)	
13–14	281,849 (31.7)	10,619 (28.1)		133,685 (12.5)	13,014 (10.8)	
15–16	408,323 (46.0)	16,873 (44.7)		417,228 (38.9)	45,148 (37.3)	
>16	159,374 (17.9)	8566 (22.7)		511,277 (47.7)	61,853 (51.1)	
**Age at menopause, years,** **mean (SD)**				49.99 (±4.0)	50.05 (±4.2)	<0.001
<40				18,050 (1.7)	2469 (2.0)	<0.001
40–44				61,482 (5.7)	7415 (6.1)	
45–49				296,664 (27.7)	31,855 (26.3)	
50–54				587,412 (54.8)	64,433 (53.2)	
≥55				109,171 (10.2)	14,930 (12.3)	
**Parity**			<0.001			<0.001
1	117,837 (13.3)	4709 (12.5)		67,094 (6.3)	5858 (4.8)	
≥2	734,191 (82.6)	31,736 (84.1)		979,503 (91.3)	112,843 (93.2)	
Nullipara	36,432 (4.1)	1291 (3.4)		26,182 (2.4)	2401 (2.0)	
**Duration of BF, months**			<0.001			<0.001
<6	219,610 (24.7)	7003 (18.6)		72,661 (6.8)	5733 (4.7)	
6–12	234,963 (26.5)	9516 (25.2)		188,313 (17.6)	18,008 (14.9)	
≥12	272,801 (30.7)	14,722 (39.0)		739,956 (69.0)	90,729 (74.9)	
Never	161,086 (18.1)	6495 (17.2)		71,849 (6.7)	6632 (5.5)	
**Total reproductive years,** **mean (SD)**				33.55 (±4.4)	33.47 (±4.6)	<0.001
<30				147,895 (13.8)	18,220 (15.1)	<0.001
<35				449,813 (41.9)	49,907 (41.2)	
<40				409,574 (38.2)	44,264 (36.6)	
≥40				65,497 (6.1)	8711 (7.2)	
**Duration of OC use, years**			<0.001			<0.001
Never	772,147 (86.9)	32,090 (85.0)		906,165 (84.5)	101,236 (83.6)	
<1	85,969 (9.7)	3922 (10.4)		101,590 (9.5)	11,447 (9.5)	
≥1	30,344 (3.4)	1724 (4.6)		65,024 (6.1)	8419 (7.0)	
**Duration of HRT, years**						<0.001
Never				893,677 (83.3)	103,099 (85.1)	
<2				104,126 (9.7)	10,369 (8.6)	
2–5				43,057 (4.0)	4058 (3.4)	
≥5				31,919 (3.0)	3576 (3.0)	

Abbreviations: OC: oral contraceptive, BF: breast feeding, HRT: hormone replacement therapy.

**Table 2:** Associations between body mass index, waist circumference, and type 2 diabetes risk by menopausal status.

		Subjects (*n*)	Events (*n*)	Duration (Person-Years)	Incidence Rate (per 1000 Person-Years)	HR (95% C.I)			
						Model 1	Model 2	Model 3	Model 4
Body Mass Index									
Pre-menopause	<18.5	26,508	237	219,317.7	1.08	0.52 (0.46, 0.60)	0.61 (0.53, 0.69)	0.62 (0.54, 0.70)	0.62 (0.55, 0.71)
	<23	463,858	7929	3,834,458.2	2.07	1 (Ref.)	1 (Ref.)	1 (Ref.)	1 (Ref.)
	<25	216,573	8598	1,775,304.8	4.84	2.35 (2.28, 2.42)	1.99 (1.93, 2.05)	1.91 (1.85, 1.97)	1.90 (1.85, 1.96)
	<30	193,705	16,195	1,557,996.3	10.40	5.07 (4.93, 5.20)	3.72 (3.62, 3.82)	3.38 (3.28, 3.47)	3.35 (3.26, 3.45)
	≥30	25,552	4777	195,232.5	24.47	12.09 (11.67, 12.53)	7.65 (7.37, 7.93)	6.25 (6.02, 6.48)	6.18 (5.95, 6.41)
	*p*-value					<0.001	<0.001	<0.001	<0.001
	*p* for trend					<0.001	<0.001	<0.001	<0.001
Post-menopause	<18.5	27,247	1346	211,878.1	6.35	0.88 (0.83, 0.92)	0.81 (0.77, 0.86)	0.87 (0.82, 0.92)	0.87 (0.83, 0.92)
	<23	425,354	24,883	3,419,151.6	7.28	1 (Ref.)	1 (Ref.)	1 (Ref.)	1 (Ref.)
	<25	317,905	29,625	2,526,788.6	11.72	1.61 (1.59, 1.64)	1.50 (1.48, 1.53)	1.44 (1.41, 1.46)	1.43 (1.41, 1.45)
	<30	379,128	54,754	2,933,978.5	18.66	2.57 (2.53, 2.61)	2.19 (2.16, 2.22)	2.00 (1.97, 2.03)	1.99 (1.96, 2.02)
	≥30	44,247	10,494	323,460.4	32.44	4.48 (4.38, 4.59)	3.41 (3.34, 3.49)	2.96 (2.89, 3.02)	2.94 (2.87, 3.01)
	*p*-value					<0.001	<0.001	<0.001	<0.001
	*p* for trend					<0.001	<0.001	<0.001	<0.001
**Waist circumference (cm)**									
Pre-menopause	<75	478,630	8121	3,955,873.2	2.05	0.42 (0.41, 0.43)	0.50 (0.48, 0.51)	0.52 (0.50, 0.53)	0.52 (0.51, 0.54)
	<80	211,629	8472	1,734,159.5	4.89	1 (Ref.)	1 (Ref.)	1 (Ref.)	1 (Ref.)
	<85	135,126	8838	1,095,861.7	8.07	1.66 (1.61, 1.71)	1.49 (1.44, 1.53)	1.44 (1.39, 1.48)	1.43 (1.39, 1.47)
	<90	62,140	6116	496,785.1	12.31	2.54 (2.45, 2.62)	2.09 (2.02, 2.16)	1.93 (1.87, 2.00)	1.92 (1.86, 1.98)
	<95	24,941	3445	195,616.5	17.61	3.65 (3.50, 3.79)	2.80 (2.69, 2.92)	2.49 (2.39, 2.59)	2.47 (2.37, 2.57)
	≥95	13,730	2744	104,013.5	26.38	5.51 (5.28, 5.75)	3.90 (3.73, 4.07)	3.27 (3.13, 3.42)	3.24 (3.10, 3.38)
	*p*-value					<0.001	<0.001	<0.001	<0.001
	*p* for trend					<0.001	<0.001	<0.001	<0.001
Post-menopause	<75	330,150	15,666	2,673,318.7	5.86	0.59 (0.57, 0.60)	0.64 (0.63, 0.66)	0.67 (0.66, 0.69)	0.67 (0.66, 0.69)
	<80	278,556	22,281	2,226,416.4	10.01	1 (Ref.)	1 (Ref.)	1 (Ref.)	1 (Ref.)
	<85	279,289	31,458	2,195,118.2	14.33	1.43 (1.41, 1.46)	1.33 (1.31, 1.36)	1.29 (1.27, 1.31)	1.29 (1.27, 1.31)
	<90	172,022	25,296	1,323,517.1	19.11	1.91 (1.88, 1.95)	1.67 (1.64, 1.70)	1.58 (1.55, 1.61)	1.58 (1.55, 1.61)
	<95	86,955	15,821	65,4781.3	24.16	2.42 (2.38, 2.47)	1.99 (1.94, 2.03)	1.84 (1.80, 1.88)	1.84 (1.80, 1.88)
	≥95	46,909	10,580	342,105.5	30.93	3.11 (3.04, 3.18)	2.38 (2.32, 2.44)	2.15 (2.10, 2.20)	2.15 (2.10, 2.20)
	*p*-value					<0.001	<0.001	<0.001	<0.001
	*p* for trend					<0.001	<0.001	<0.001	<0.001

Model 1: non-adjusted. Model 2: adjusted for age, income, smoking, alcohol drinking, regular physical activity, and glucose level. Model 3 adjusted for age, income, smoking, alcohol drinking, regular physical activity, hypertension, hyperlipidemia, chronic kidney disease, and glucose level. Model 4 (premenopausal) adjusted for age, income, smoking, alcohol drinking, regular physical activity, hypertension, hyperlipidemia, chronic kidney disease, duration of oral contraceptive use, parity, duration of breastfeeding, age of menarche, and glucose level. Model 4 (postmenopausal) adjusted for age, income, smoking, alcohol drinking, regular physical activity, hypertension, hyperlipidemia, chronic kidney disease, duration of oral contraceptive use, parity, duration of breastfeeding, age of menopause, duration of hormonal replacement therapy, and glucose level.

**Table 3:** Associations between body mass index, waist circumference, and type 2 diabetes risk by age group.

		Subjects (*n*)	Events (*n*)	Duration (Person-Years)	Incidence Rate (per 1000 Person-Years)	HR (95% C.I)		
Body Mass Index						Model 1	Model 2	Model 3
Age 40–49	<18.5	25,864	228	213,778.1	1.07	0.57 (0.50, 0.65)	0.64 (0.42, 0.98)	0.66 (0.43, 1.02)
	<23	426,550	6640	3,526,027.9	1.88	1 (Ref.)	1 (Ref.)	1 (Ref.)
	<25	188,091	6864	1,542,779.7	4.45	2.37 (2.29, 2.45)	2.09 (1.86, 2.36)	1.97 (1.75, 2.22)
	<30	163,509	12,653	1,317,875.7	9.60	5.14 (4.99, 5.29)	3.54 (3.18, 3.94)	3.17 (2.85, 3.54)
	≥30	21,806	3899	167,153.0	23.33	12.67 (12.17, 13.18)	7.61 (6.54, 8.84)	6.15 (5.27, 7.17)
	*p*-value					<0.001	<0.001	<0.001
	*p* for trend					<0.001	<0.001	<0.001
Age 50–59	<18.5	10,953	233	90,262.1	2.58	0.56 (0.49, 0.63)	0.60 (0.52, 0.69)	0.64 (0.55, 0.73)
	<23	254,769	9720	2,093,159.6	4.64	1 (Ref.)	1 (Ref.)	1 (Ref.)
	<25	176,462	12,209	1,431,781.9	8.53	1.84 (1.79, 1.89)	1.64 (1.59, 1.69)	1.56 (1.52, 1.61)
	<30	184,147	22,235	1,457,318.4	15.26	3.30 (3.22, 3.38)	2.59 (2.52, 2.66)	2.35 (2.29, 2.42)
	≥30	21,554	5025	160,396.5	31.33	6.85 (6.61, 7.08)	4.57 (4.40, 4.75)	3.84 (3.69, 4.00)
	*p*-value					<0.001	<0.001	<0.001
	*p* for trend					<0.001	<0.001	<0.001
Age ≥60	<18.5	16,938	1122	127,155.6	8.82	0.88 (0.82, 0.93)	0.86 (0.81, 0.91)	0.91 (0.86, 0.97)
	<23	207,893	16,452	1,634,422.3	10.07	1 (Ref.)	1 (Ref.)	1 (Ref.)
	<25	169,925	19,150	1,327,531.8	14.43	1.43 (1.40, 1.46)	1.39 (1.36, 1.42)	1.33 (1.30, 1.36)
	<30	225,177	36,061	1,716,780.6	21.01	2.09 (2.05, 2.13)	1.91 (1.87, 1.94)	1.77 (1.74, 1.80)
	≥30	26,439	6347	191,143.4	33.21	3.31 (3.21, 3.40)	2.75 (2.67, 2.83)	2.44 (2.36, 2.51)
	*p*-value					<0.001	<0.001	<0.001
	*p* for trend					<0.001	<0.001	<0.001
**Waist circumference (cm)**								
Age 40–49	<75	441,948	6887	3,652,464.9	1.89	0.42 (0.40, 0.43)	0.48 (0.42, 0.54)	0.50 (0.45, 0.57)
	<80	184,815	6849	1,515,174.3	4.52	1 (Ref.)	1 (Ref.)	1 (Ref.)
	<85	114,722	6878	931,954.0	7.38	1.64 (1.58, 1.69)	1.42 (1.26, 1.60)	1.37 (1.22, 1.55)
	<90	52,003	4792	416,527.5	11.51	2.56 (2.47, 2.66)	1.93 (1.69, 2.20)	1.79 (1.57, 2.04)
	<95	20,804	2679	163,718.1	16.36	3.66 (3.50, 3.83)	2.57 (2.20, 3.01)	2.29 (1.95, 2.68)
	≥95	11,528	2199	87,775.5	25.05	5.66 (5.39, 5.94)	3.48 (2.90, 4.17)	2.91 (2.42, 3.49)
	*p*-value					<0.001	<0.001	<0.001
	*p* for trend					<0.001	<0.001	<0.001
Age 50–59	<75	230,613	7814	1,898,605.6	4.12	0.53 (0.51, 0.54)	0.59 (0.58, 0.61)	0.62 (0.60, 0.64)
	<80	164,648	10,467	1,338,453.7	7.82	1 (Ref.)	1 (Ref.)	1 (Ref.)
	<85	134,782	12,715	1,080,880.5	11.76	1.51 (1.47, 1.55)	1.38 (1.34, 1.42)	1.34 (1.30, 1.38)
	<90	70,762	9377	556,687.1	16.84	2.16 (2.10, 2.23)	1.86 (1.80, 1.92)	1.75 (1.69, 1.80)
	<95	31,062	5355	239,324.6	22.38	2.88 (2.79, 2.98)	2.29 (2.21, 2.38)	2.10 (2.02, 2.18)
	≥95	16,018	3694	118,967.1	31.05	4.02 (3.87, 4.17)	2.93 (2.81, 3.05)	2.57 (2.47, 2.68)
	*p*-value					<0.001	<0.001	<0.001
	*p* for trend					<0.001	<0.001	<0.001
Age ≥60	<75	136,219	9086	1,078,121.3	8.43	0.69 (0.68, 0.71)	0.72 (0.70, 0.74)	0.75 (0.73, 0.77)
	<80	140,722	13,437	1,106,947.8	12.14	1 (Ref.)	1 (Ref.)	1 (Ref.)
	<85	164,911	20,703	1,278,145.4	16.20	1.34 (1.31, 1.36)	1.28 (1.26, 1.31)	1.25 (1.22, 1.28)
	<90	111,397	17,243	847,087.6	20.36	1.68 (1.64, 1.72)	1.55 (1.52, 1.59)	1.48 (1.45, 1.52)
	<95	60,030	11,232	447,355.1	25.11	2.07 (2.02, 2.12)	1.83 (1.79, 1.88)	1.72 (1.68, 1.76)
	≥95	33,093	7431	239,376.5	31.04	2.56 (2.49, 2.64)	2.15 (2.09, 2.21)	1.98 (1.92, 2.04)
	*p*-value					<0.001	<0.001	<0.001
	*p* for trend					<0.001	<0.001	<0.001

Model 1: non-adjusted. Model 2: adjusted for age, income, smoking, alcohol drinking, regular physical activity, and glucose level. Model 3 adjusted for age, income, smoking, alcohol drinking, regular physical activity, hypertension, hyperlipidemia, chronic kidney disease, and glucose level.

**Table 4:** Associations between body mass index, waist circumference, and type 2 diabetes risk by menopausal status in 45–54-year-old women.

		Subjects (*n*)	Events (*n*)	Duration (Person-Years)	Incidence Rate (per 1000 Person-Years)	HR (95% C.I)			
						Model 1	Model 2	Model 3	Model 4
**Body mass index**									
Pre Menopause	<18.5	8361	111	69,224.9	1.60	0.56 (0.46, 0.67)	0.60 (0.50, 0.73)	0.62 (0.52, 0.75)	0.63 (0.52, 0.76)
	<23	200,973	4779	1,661,144.1	2.88	1 (Ref.)	1 (Ref.)	1 (Ref.)	1 (Ref.)
	<25	109,860	5289	899,213.0	5.88	2.05 (1.97, 2.13)	1.84 (1.77, 1.92)	1.76 (1.69, 1.83)	1.75 (1.68, 1.82)
	<30	101,199	9821	810,970.4	12.11	4.24 (4.10, 4.39)	3.37 (3.25, 3.49)	3.04 (2.93, 3.14)	3.01 (2.90, 3.12)
	≥30	12,212	2592	92,367.6	28.06	9.97 (9.50, 10.46)	6.75 (6.43, 7.08)	5.44 (5.18, 5.72)	5.37 (5.11, 5.65)
	*p* for trend					<0.001	<0.001	<0.001	<0.001
Post Menopause	<18.5	4931	84	40,632.6	2.07	0.55 (0.44, 0.68)	0.57 (0.46, 0.71)	0.60 (0.48, 0.74)	0.60 (0.49, 0.75)
	<23	93,942	2906	773,287.6	3.76	1 (Ref.)	1 (Ref.)	1 (Ref.)	1 (Ref.)
	<25	53,503	3302	434,935.5	7.59	2.03 (1.93, 2.13)	1.83 (1.74, 1.92)	1.74 (1.66, 1.83)	1.74 (1.65, 1.83)
	<30	49,486	5362	393,467.8	13.63	3.65 (3.49, 3.82)	2.98 (2.85, 3.12)	2.69 (2.57, 2.81)	2.68 (2.56, 2.81)
	≥30	5582	1230	41,681.2	29.51	7.98 (7.46, 8.53)	5.61 (5.24, 6.00)	4.57 (4.27, 4.90)	4.57 (4.26, 4.89)
	*p* for trend					<0.001	<0.001	<0.001	<0.001
***p* for interaction** **(menopause vs. BMI)**						<0.001	<0.001	<0.001	
**Waist circumference (cm)**									
Pre Menopause	<75	203,936	4728	1,685,743.7	2.81	0.47 (0.45, 0.48)	0.52 (0.50, 0.54)	0.55 (0.53, 0.57)	0.55 (0.53, 0.57)
	<80	106,004	5220	867,130.1	6.02	1 (Ref.)	1 (Ref.)	1 (Ref.)	1 (Ref.)
	<85	70,574	5406	570,947.6	9.47	1.58 (1.52, 1.64)	1.46 (1.40, 1.51)	1.40 (1.35, 1.46)	1.40 (1.34, 1.45)
	<90	32,471	3733	258,162.9	14.46	2.42 (2.32, 2.52)	2.07 (1.98, 2.15)	1.90 (1.82, 1.98)	1.89 (1.81, 1.97)
	<95	13,010	2033	101,274.9	20.07	3.37 (3.21, 3.55)	2.66 (2.53, 2.81)	2.36 (2.24, 2.49)	2.34 (2.22, 2.47)
	≥95	6610	1472	49,660.8	29.64	5.02 (4.74, 5.32)	3.65 (3.44, 3.87)	3.05 (2.87, 3.23)	3.02 (2.85, 3.20)
	*p* for trend					<0.001	<0.001	<0.001	<0.001
Post Menopause	<75	88,958	2516	732,961.0	3.43	0.49 (0.47, 0.52)	0.54 (0.52, 0.57)	0.57 (0.54, 0.60)	0.57 (0.54, 0.60)
	<80	51,470	2917	419,250.9	6.96	1 (Ref.)	1 (Ref.)	1 (Ref.)	1 (Ref.)
	<85	37,343	3128	300,501.7	10.41	1.50 (1.43, 1.58)	1.40 (1.33, 1.48)	1.35 (1.29, 1.43)	1.36 (1.29, 1.43)
	<90	18,178	2226	143,549.5	15.51	2.24 (2.12, 2.37)	1.92 (1.82, 2.03)	1.79 (1.69, 1.89)	1.79 (1.69, 1.89)
	<95	7610	1267	58,641.4	21.61	3.13 (2.93, 3.35)	2.54 (2.38, 2.71)	2.28 (2.14, 2.44)	2.28 (2.13, 2.44)
	≥95	3885	830	29,100.3	28.52	4.16 (3.85, 4.49)	3.12 (2.88, 3.37)	2.66 (2.46, 2.87)	2.65 (2.45, 2.87)
	*p* for trend					<0.001	<0.001	<0.001	<0.001
***p* for interaction** **(menopause vs. WC)**						**<0.001**	**<0.001**	**<0.001**	

Model 1: non-adjusted. Model 2: adjusted for age, income, smoking, alcohol drinking, regular physical activity, and glucose level. Model 3 adjusted for age, income, smoking, alcohol drinking, regular physical activity, hypertension, hyperlipidemia, chronic kidney disease, and glucose level. Model 4 adjusted for age, income, smoking, alcohol drinking, regular physical activity, hypertension, hyperlipidemia, chronic kidney disease, duration of oral contraceptive use, parity, duration of breastfeeding, age of menopause, duration of hormonal replacement therapy, and glucose level.

## Data Availability

The data presented in this study could be accessed via the Health Insurance Data Service website (http://nhiss.nhis.or.kr). The raw data cannot be retrieved from the server unless the researchers submit a study proposal for acquiring approval from each institutional review board, which is also reviewed by the NHIS review committee to access to the database.
